# Clathrin recruitment and assembly of phosphorylated B cell receptor is impaired in human diffuse large B cell lymphoma

**DOI:** 10.64898/2026.09.22.753512

**Published:** 2026-09-23

**Authors:** Aleah D. Roberts, Kem A. Sochacki, Louis M. Staudt, Justin W. Taraska

## Abstract

The B cell receptor (BCR) drives the differentiation of naive B cells into activated plasma cells that produce antibodies. The BCR consists of a plasma membrane-bound surface immunoglobulin (sIg) that binds antigens, and two coreceptor subunits CD79A and CD79B. The intracellular domains of CD79A and CD79B initiate immune signaling and endocytosis of the receptor in healthy B cells. In diffuse large B cell lymphoma (DLBCL), an aggressive form of human blood cancer, the activated B cell like (ABC) subtype exhibits constitutive signaling that drives survival and proliferation. Here, in a model of human ABC DLBCL, we determine the localization of BCR components relative to plasma membrane structures using correlative super-resolution light and platinum replica transmission electron microscopy. We find that spontaneous clusters of the surface Immunoglobulin common to ABC lymphoma localize to smooth raised membrane domains. These structures are involved in the endocytosis of large receptor clusters. Surprisingly, activated phosphorylated CD79A (pCD79A) shows little colocalization with clathrin on or off smooth raised membranes. Furthermore, pCD79A spatially segregates away from both the slg and CD79B subunits of the BCR complex. A similar distribution was found for downstream phosphorylated Src family kinases. We propose that a disengagement of pCD79A away from sIg and CD79B allows the receptor to evade down-regulation through endocytosis and maintains signaling at the plasma membrane. This mechanism could drive aberrant signaling and the proliferation of lymphomas leading to disease

## Full Text Availability

The license terms selected by the author(s) for this preprint version do not permit archiving in PMC. The full text is available from the preprint server.

